# Supplementary material for: Are carbon emissions trading and green financial instruments synergistic? -Comprehensive quantitative research based on content analysis
Source: PLoS One. 2024 Mar 7;19(3):e0298601. doi: 10.1371/journal.pone.0298601 (PMC10919601; doi:10.1371/journal.pone.0298601)
Supplement: S1 Data — (ZIP) [file pone.0298601.s001.zip › Data/地级市Z_F工作报告.zip!/║╙─╧/╨φ▓2╩╨/└┤╫╘╣1⁄2╓┌║┼í╛┬φ┐╦╩2╛▌═°í┐_2010.docx]

各位代表：

现在，我代表市人民政府向大会作工作报告，请予审议，并请市政协各位委员提出意见。

一、2009年工作回顾

2009年，是新世纪以来我市经济发展最为困难的一年，也是我们砥砺奋进，战胜严峻考验，取得重大成绩的一年。面对国际金融危机严重冲击，在省委、省政府和市委的正确领导下，全市上下深入贯彻落实科学发展观，迎难而上，顽强拼搏，积极应对，较好地完成了市五届人大四次会议确定的各项任务，取得了化危为机、跨越发展的重大胜利。

——经济形势回升向好。全市生产总值完成1176.2亿元，增长12.5%；地方财政一般预算收入47.1亿元，增长13.1%，境内财政总收入126.3亿元，增长17.2%，“双11%”增长目标圆满实现。

——项目建设成效显著。100个亿元以上重点项目完成投资225亿元，带动全社会固定资产投资678.4亿元，增长30%，投资规模、建设速度为历年之最。

——结构调整扎实推进。全市规模以上工业增加值完成562.3亿元，增长17.9%。第三产业增速分别高于全国、全省4.4和2.4个百分点，三次产业结构调整为11.6：66：22.4。城镇化率达到39.3%，提高1.75个百分点。

——人民生活持续改善。“十大实事”全面完成。全市涉及民生支出55.2亿元，占一般预算支出的55%，增长41.3%。城镇登记失业率控制在3.2%。城镇居民人均可支配收入13619元，农民人均纯收入6302元，分别增长9.4%和7.9%。

一年来，我们突出抓了以下重点工作：

（一）立足扩大内需，促进经济增长。面对严峻经济形势，战危机、保增长，及时出台应对之策，全市生产总值增速从一季度的7.8%逐步回升到全年的12.5%。针对投资不振，实施“3816”投资促进计划，建立重点项目联动和资源配置机制，争取中央新增投资6.34亿元，禹州电厂二期、泉店煤矿、青山金汇不锈钢三期、许昌500KV变电站等37个项目竣工投产，许继风电产业园、许昌航空港产业园、石武客专许昌段等51个项目开工建设。针对企业不稳，实施“重点服务800家企业计划”，减轻企业负担5.4亿元，投放贷款183亿元，利用资本市场融资50亿元。针对消费不旺，实施一系列扩大消费需求措施，落实家电、汽车摩托车下乡、汽车以旧换新政策，社会消费品零售总额297亿元，增长19.5%，旅游总收入增长28%，商品房销售面积增长42.7%。

（二）转变发展方式，推动转型升级。中原电气谷建设全面铺开，10个产业集聚区实现主营业务收入830亿元，增长20%，利税72.1亿元，增长21%，集聚产业、支撑发展、示范带动作用不断增强。编制实施电力电子装备、食品、发制品和电动汽车产业发展规划，3大主导产业增加值占GDP的25%，许继集团销售收入超百亿元。54个重大工业结构调整项目完成投资63亿元。实施自主创新体系建设和发展规划，3家企业被认定为国家级创新型和创新型试点企业，新增国家级高新技术企业和企业研发中心5个，获得国家科学技术进步一等奖1项，荣获“全国科技进步先进市”，科技进步对经济增长的贡献率达到53%。大力推进节能减排，单位GDP能耗下降4%，化学需氧量、二氧化硫排放量分别下降7.54%和15.6%，环境空气质量优良天数达到329天。

（三）统筹城乡发展，夯实“三农”基础。认真落实强农惠农政策，重灾之年粮食再夺丰收，总产273万吨，连续6年创历史新高。编制实施花卉苗木产业发展振兴规划，花木、蔬菜、中药材种植面积分别达到90、100和40万亩。畜牧业总产值110亿元，占农业总产值的46.1%。完成8万亩中低产田改造和10座中小水库除险加固任务，新增有效灌溉面积20万亩。新增农村户用沼气6万口，改建农村公路638公里，解决22万人饮水安全问题。新建农村连锁超市100家。集体林地完成确权150.9万亩，土地流转面积48.5万亩，新增农民专业合作组织216家。完成18个扶贫开发重点村整村推进任务，1.3万人脱贫。许昌新区建设总体方案获得省委、省政府批准，开工建设190个涉及基础设施、产业发展、社会事业项目，完成投资87亿元，加快实施 18个城中村改造项目，教育、医疗、卫生、社会保障等项事业协调发展，许昌新区已经成为全市经济社会发展中的崭新亮点。

（四）坚持城市带动，提升建管水平。充分发挥规划引领作用，市区重点地段控制性详规实现全覆盖，5个县（市）村镇体系规划、47个乡（镇）总体规划修编和283个村庄规划全面完成，城乡规划体系不断完善。75个城建重点项目竣工投用，完成投资14.6亿元。灞陵路中段、许都路中段建成通车，八一路、仓库路改造升级，天宝河游园、学院河游园建成开放。市会展中心竣工投用，市文博馆、体育馆加快建设。35个城中村拆迁327万平方米，完成投资37.6亿元。城市管理精细化、数字化和夜景灯饰化扎实推进。新建污水处理厂3个，饮用水源地保护全面加强，“创模”工作取得新进展。荣获全国绿化模范城，文明城市创建实现新突破，“四城同创”成果不断巩固。城市规划、建设和管理水平持续提升。

（五）深化改革开放，增强发展活力。40家企事业单位完成改革改制任务。市县政府机构改革进展顺利。教育、医疗、卫生、文化等重点领域改革扎实推进。“1253”大招商活动成效显著，签约项目284个，到位资金155.8亿元。与国家电网、中国平安、中烟公司、郑州国际机场等大型企业的战略合作卓有成效。组建市投资总公司和8个产业集聚区投融资平台，融资34.2亿元。森源电气成功上市，交通银行顺利入驻，光大银行批准入驻，市商业银行更名升级为许昌银行，成为全国性商业银行，第一家农村商业银行成立，新增担保机构14家，荣获“中国金融生态城市”称号。成功举办第六届中国花木产业高峰论坛、第九届中原花木交易博览会、中国许昌第三届三国文化周和第六届钧瓷文化旅游节，对外开放水平进一步提升。进出口总值11.1亿美元，增长24.3%；其中出口8.6亿美元，增长25.2%，出口总量和增速均居全省第2位。

（六）切实改善民生，促进和谐稳定。加大国家级创业型城市创建力度，新增各类经济组织2.4万家，新增城镇就业11.9万人、农村劳动力转移就业16.2万人、下岗失业人员再就业2.5万人，城镇“零就业家庭”保持动态为零。“两免一补”政策全面落实，维修改造农村中小学校舍4.3万平方米，新建校舍5.5万平方米，职业教育攻坚、市区教育资源整合扎实推进，许昌技师学院开工建设，许昌陶瓷职业学院获省批准。建成乡镇综合文化站10个、农家书屋190个、文化信息资源共享工程村级服务点922个。开工建设东城区医院，改造乡镇卫生院25个、标准化村卫生室570个。城镇职工和居民基本医疗保险覆盖面不断扩大，新农合参合率达到94.99%，补偿参合农民23万人次。甲型H1N1流感和手足口病疫情得到有效防控。企业退休人员基本养老金人均月增120元，新型农村养老保险试点工作扎实推进，农村五保集中供养率达到60.8%，居全省前列。新建廉租住房7.7万平方米，经济适用房25.5万平方米，增长40.9%。人口自然增长率控制在4.3‰。

（七）加强政府建设，推进民主法治。坚持依法行政，自觉接受人大及其常委会的监督，支持政协参政议政，办理人大代表议案建议、政协委员提案347件。深入开展学习实践科学发展观活动，扎实推进“五个坚持，两转两提”，大力倡树清新、简约、责实、务本的良好政风。深化政务公开，市政府网站公开信息9056条，市长信箱来信办结率99.98%。深化财政投资评审，审减资金2.4亿元，审减率达19%。加强社会治安综合治理，扎实做好信访稳定工作，荣获“全省平安建设先进省辖市”。切实加强煤炭等行业安全监管，安全生产形势总体平稳。加强政府廉政建设，深化财政审计监督，反腐倡廉取得新成效。军政军民更加团结，荣获“全省双拥模范城”。民族、宗教、外事、侨务、对台工作全面加强，荣获“全国民族团结进步模范集体”。援助四川江油灾后重建工作进展顺利。地方史志、档案、统计、体育、地震、气象等工作都取得了新的成绩。

各位代表，回顾过去一年，我们在应对危机的重大考验中，深化了对坚持科学发展、促进和谐稳定的认识，积累了在复杂形势下推动经济社会发展的宝贵经验。一是必须做到保增长与调结构相统一，让发展更协调、更持续。二是必须做到抓好薄弱环节与谋划长远发展相统一，为科学发展奠定基础。三是必须做到改革创新与扩大开放相统一，不断破解发展难题，增强发展活力。四是必须做到扩大投资与改善民生相统一，让经济增长的动力更持久、发展的成果更惠民。五是必须做到增强政府驾驭复杂局面的能力与提升运作水平相统一，科学决策，注重运作，求实求效。

各位代表，过去一年的成绩着实来之不易。能够在应对危机中持续开创新的局面，得益于省委、省政府和市委的坚强领导，得益于人大、政协和社会各界的大力支持，得益于全市人民的团结奋斗。在此，我代表市人民政府，向全市广大干部群众、驻许部队、武警部队官兵和政法干警表示崇高的敬意！向所有关心、支持许昌建设与发展的社会各界人士表示衷心的感谢！

在肯定成绩的同时，我们也清醒地看到还存在不少的困难和问题，按照科学发展观的要求，有些尤为突出。一是经济增长内生动力不强，自主创新能力不足，要素制约逐步趋紧。二是经济结构不优，服务业比重和水平较低，城镇化率偏低，统筹城乡发展任务艰巨。三是支撑作用强的重大项目较少，项目运作水平不高。四是民生改善不够，社会事业欠账较多，一些涉及群众切身利益的问题还没有得到很好解决。五是政府自身建设有待加强，职能转变不到位，执行力不强，官僚主义、形式主义仍然存在，腐败现象时有发生。对此，我们要高度重视，坚定信心，认真解决。

二、2010年总体要求和主要目标

今年是继续保持经济平稳较快发展、加快经济发展方式转变的关键一年，是全面实现“十一五”规划目标、为“十二五”发展打好基础的重要一年。无论从国际、国内，还是许昌的情况来看，各种积极因素和不利影响此消彼长，机遇与挑战并存共生，形势极为复杂。对此，我们必须全面、正确地判断形势，既要增强机遇意识，坚定必胜信心，又要保持清醒头脑，增强忧患意识，始终把握工作主动权。

今年政府工作的总体要求：全面贯彻党的十七大和十七届三中、四中全会精神，按照中央、省委和市委的决策部署，深入贯彻落实科学发展观，以“一争四推三提升”为统领，以加快经济发展方式转变为工作主线，以持续保持经济平稳较快发展为主要任务，以改革开放、自主创新为根本动力，以改善民生、促进和谐为根本目标，加快推进新型工业化、特色城镇化、农业现代化，全面完成“十一五”规划各项目标任务，努力实现经济社会又好又快发展。

今年经济社会发展的主要预期目标：地区生产总值确保增长11%、力争12%，地方财政一般预算收入增长12%，全社会固定资产投资增长21%，社会消费品零售总额增长16%，外贸出口增长12%，实际利用外商直接投资增长16%，居民消费价格涨幅控制在3%左右，城镇居民人均可支配收入实际增长7%，农民人均纯收入实际增长6%，人口自然增长率控制在6.3‰以内，城镇新增就业5万人，登记失业率控制在4%以内，万元生产总值能耗下降3.5%，化学需氧量、二氧化硫排放量分别控制在1.29万吨和3.4万吨以下。

实现上述目标，做好今年工作，要始终坚持“重在持续、重在提升、重在统筹、重在为民”，把握好以下几个方面：一是把重持续与促转变结合起来，使二者并重。持续发展的意识、发展的思路、发展的进程，持续保持经济较快增长，更加注重结构调整，加快经济发展方式转变，不断赢得更大的发展空间。二是把重投资与促消费结合起来，使二者共推。不断优化投资结构，保持投资较快增长，着力扩大消费需求，寻求出口潜力，增强消费和出口的拉动作用，催生发展的内生动力。三是把重发展与惠民众结合起来，使二者统一。转变发展的目的，在促进发展中坚持重在为民，围绕重在为民促进发展，着力改善民生、发展社会事业，让发展成果由人民共享。四是把重即期与谋长远结合起来，使二者同步。在保增长的同时，着眼于抢占下一轮发展的制高点，重运作、重统筹、重谋划，多做打基础、利长远的事，让发展的基础越来越坚实。五是把重提升与强服务结合起来，使二者协同。提升执行能力，提高行政效能，增强服务意识，创新服务手段，让政务环境更优，让创业氛围更浓，使许昌在科学发展的道路上迈出更加铿锵有力的坚实步伐。

三、努力保持经济平稳较快发展

坚持项目带动，立足扩大内需，培育壮大市场主体，不断拉动经济增长。

（一）持续保持投资快速增长。更加注重优化投资结构，大力实施“3618”投资促进计划，围绕基础产业、基础设施、基础要素3大基础，在工业、服务业、社会事业、城市基础设施和城中村改造、农林水利、交通等6个领域，实施100个投资亿元以上的重点项目，带动全社会固定资产投资800亿元。工业方面，大力实施风力发电机组、风电研发中心、风电齿轮箱、智能电网、轨道交通智能型中压开关、西继电梯生产基地等装备制造业项目，积极推进新区热电厂、帝豪专线、森源集团微型电动车、新能源汽车零部件、瑞贝卡纤维发丝三期工程、康得药业迁建工程等60个重点项目，完成投资100亿元。现代服务业方面，加快建设许昌航空港产业园、金融通信服务提升工程、深国投商业中心（沃尔玛）、众品生鲜食品冷链物流园等16个重点项目，完成投资23.2亿元；加快推进市旅游服务中心、灞陵桥景区综合开发等11个文化旅游产业重点项目，完成投资7.8亿元。社会事业方面，市文博馆、市体育馆、人民医院综合住院楼等项目竣工投用，许昌技师学院一期工程完工，东城区医院、许昌学院扩建工程等项目加快推进，许昌广电中心、许昌创业服务中心等项目开工建设，完成投资13.5亿元。农林水利方面，开工建设南水北调中线工程及配套设施，大力实施生态林业提升工程、鄢陵名优花木生产科技园区、鲜切花温室基地等重点项目，完成投资14.3亿元。交通方面，许禹铁路改造一期工程、京珠高速许昌段拓宽改造、永登高速许昌连接线、311国道许昌至鄢陵段改建工程等项目竣工通车，加快建设石武客专许昌段，开工建设安信公路许昌段和郑州机场至许昌城际铁路，完成投资34亿元。切实提升项目运作水平，着力谋划、包装、储备、落地一批事关全局的重大项目，持续增强发展后劲。

（二）积极扩大居民消费需求。落实完善扩大消费的政策措施，真正让广大群众得到实惠。继续提高农民收入、企业退休人员基本养老金和部分优抚对象待遇，城市低保标准提高到每人每月不低于190元，农村低保标准提高到每人每年不低于1100元，增强居民特别是中低收入者的消费能力。抓好社区商业、农贸市场改造升级和早餐工程，积极培育信息、文化、旅游、养老、家庭服务等消费热点。继续实施家电、汽车摩托车下乡和汽车以旧换新政策。加快万村千乡市场工程、新网工程建设，新建农家店110家、农村标准超市100个。整顿和规范市场秩序，加强市场价格监管，营造良好的消费环境。

（三）切实提高企业服务水平。建立服务企业长效机制，突出解决生产要素保障等问题，着力促进企业转型升级、战略合作、壮大市场主体。抓好809家重点企业服务工作，完善分包服务制度。拓宽融资渠道，推进银企对接，解决好企业特别是中小企业融资难问题，力争新增贷款120亿元。加强经济运行监测分析，保障煤电油气运供应，继续做好企业治乱减负工作，提高为企业服务的效率和水平。

四、切实加快经济发展方式转变

许昌已经进入只有加快转变经济发展方式，才能确保经济持续快速发展的关键时期，必须加快推动经济进入创新驱动、内生增长的发展轨道。

（一）加快产业集聚区建设。全面实施产业集聚区两年提升推进计划，加快建设“一带十区二十个产业集群”，致力打造区域经济新的增长极。10个产业集聚区各项规划全面完成，新上亿元以上项目79个，完成固定资产投资250亿元，实现主营业务收入1000亿元。围绕主导特色产业，完善产业链，提升附加值，扩大集群效应。强力实施中原电气谷“2415”项目攻坚计划，加快建设20个以上产业发展项目、４个园区、1个中心和5条道路。加快建设产业集聚区水电路、标准厂房等基础设施，积极发展现代物流、中介服务、技术研发以及文化教育、医疗卫生、公寓餐饮等服务业，不断完善功能。创新管理机制，加强投融资平台建设，建立健全考评体系，促进全市产业集聚区竞相发展。

（二）促进产业结构优化升级。认真落实食品、电力电子装备、发制品、电动汽车等产业发展规划，编制实施现代物流、医药产业、新兴能源等专项发展规划，提升3大主导产业、4大特色产业发展水平。推进信息化与工业化融合，改造提升传统产业，培育具有核心竞争力的电子信息产业。加快实施“千百亿工程”，对重点企业、“小巨人”企业、高新技术企业动态管理、大力扶持，力争2—3家企业销售收入突破100亿元。加快发展以风电整机为抓手的新能源、以电动汽车为抓手的新能源汽车、以人造金刚石为抓手的新材料、以智能电网为抓手的新技术、以仓储物流为抓手的现代服务业，积极培育战略性新兴产业。加快发展服务业，积极扶持20户重点服务业企业。大力发展物流、信息、研发、商务等面向生产的服务业。大力发展市政公用、房地产和物业服务、社区服务等面向民生的服务业。加快发展文化旅游业，培育市场主体，开发新型业态，致力打造“三张名片”。加快发展金融业，支持许昌银行跨区经营，积极推进光大银行、浦发银行、民生银行等金融、证券和保险机构入驻许昌。

（三）切实提升自主创新能力。加快构建以企业为主体、市场为导向、产学研相结合的技术创新体系，科技进步对经济增长的贡献率达到54%。新培育国家级高新技术企业5家，争创一批省级创新型企业，培育一批创新型科技领军人才和团队。积极推进创新型产业集聚区建设，新建20个省、市级企业研发中心。加快实施重大科技专项，不断加大科技投入，加强知识产权工作，加速科技成果转化。大力开展“质量兴市”和“品牌兴业”活动，健全质量管理体系，坚持走以质取胜之路。

（四）大力推进节能减排。加快淘汰落后产能，积极推进一批重点节能工程建设。加快建设庞庄生活垃圾综合处理厂2期、龙岗电厂中水回用工程、青浦合金脱硫改造等环保基础设施。加强饮用水源地保护，继续实施城区水系综合治理。编制实施农村环境综合整治规划，积极创建省级生态乡镇和生态文明村。实行最严格的耕地保护制度和节约用地制度，提高土地综合利用效率。编制实施低碳经济发展规划，努力建设以低碳排放为特征的产业体系和消费模式。积极发展新能源和可再生能源，争创国家再生能源建筑应用示范城市。加强林业生态建设，增加森林碳汇，确保提前2年完成林业生态市建设目标。

五、夯实“三农”发展基础

坚持稳粮保供给、增收惠民生、改革促统筹、强基增后劲，增强农业综合生产能力，提高农业现代化水平。

（一）稳定发展粮食生产。积极推进粮食生产核心区建设，稳定面积、主攻单产、提高品质、提升总产，力争粮食总产达到275万吨以上。大力开展高产创建活动，加快40个粮食高产示范方建设。加强农业综合开发，完成中低产田改造和高标准农田建设8万亩。提高粮食最低收购价，小麦每斤提高3分钱。认真落实粮食直补、农资综合直补、良种补贴和农机具购置补贴等支农惠农政策，引进优良品种，推广农业科技，提高农机社会化服务水平。

（二）大力发展现代农业。加快发展特色农业、生态农业、有机农业和循环农业。花木、蔬菜、中药材种植面积分别达到或稳定在100万亩、100万亩和40万亩。认真落实花卉苗木产业发展振兴规划，新增高档花木7万亩、鲜切花1万亩。积极推进现代烟田建设。加强畜牧业基地建设，力争畜牧业产值占农业总产值的比重达到46.5%。促进农产品加工业集群发展，新培育市级以上农业产业化龙头企业40家，新发展农民专业合作组织100家。

（三）加快完善基础设施。财政支出优先支持农业农村发展，引导和推动资源要素向农村配置。继续按照“饮干净水、走平坦路、建沼气池、用卫生厕”的要求，完善配套，提升水平，新解决农村居民安全饮水20万人，改建提升农村公路400公里，新增户用沼气5万口，改厕5800个。实施新农村建设“百村示范”工程，加快村庄规划和村容村貌综合治理。加强水库除险加固和农田水利基本建设，新打配机井1000眼，发展节水灌溉10万亩，治理水土流失30平方公里。完成南水北调中线工程第一批移民安置任务，启动第二批移民安置工作。加快农村电网改造升级。加大扶贫开发力度，再解决1.5万人脱贫问题。

（四）继续深化农村改革。落实农村基本经营制度，稳定土地承包关系。坚持“依法、自愿、有偿”，推进土地流转达到120万亩。完成集体林权制度主体改革任务，林权证发放率60%以上。积极稳妥推进以土地承包经营权置换社会保障、以宅基地置换城镇住房试点工作。加快县级农村信用社改制步伐，积极培育村镇银行、小额贷款公司等新型农村金融机构，切实改善农村金融服务。

六、加快推进城镇化进程

以城镇化为战略抓手，扩大内需，调整结构，加快经济发展方式转变，努力拓展经济增长和市场需求的新空间。

（一）全面推进许昌新区建设。认真落实许昌新区建设总体方案，按照“8个高水准”和“大发展，上水平，做示范，求实效”的要求，加快推进“三个集中，六个一体”。高标准完成许昌新区总体规划及各类配套规划，实现核心区控制性详细规划全覆盖。大力实施173个重点项目，完成投资158亿元。加快主导特色产业集群发展，做精做优花木产业和现代农业，协同发展生态旅游业，增强产业支撑。加快魏武大道、创业大道等一批基础设施建设，加大“三村”改造和新型社区建设力度，完善综合功能，提升承载能力。大力发展教育、文化、卫生、体育等公共服务事业，健全社会保障体系，3年内将许昌新区9万农村人口全部转为城镇人口。

（二）积极构建现代城镇体系。坚持“带状城市、向心布局、组团发展、城乡统筹”，着力打造“一中心五组团”，加快构建市区、新区、县（市）城区、中心镇协调发展的现代城镇体系，力争城镇化率增长2个百分点。发展壮大县域经济，加快县城、中心镇基础设施建设，鼓励返乡农民工就地创业，积极引导非农产业和农村人口向县城和城镇转移。推进城镇户籍制度改革，逐步把县城发展成为20万以上人口的中等城市，辐射带动农村发展，促进城镇化与新农村建设良性互动。

（三）全面提升城市建管水平。全面完成市区重点区域专项规划、乡镇规划和中心村规划。开工建设北外环东段、许都路东段、解放路南段、文化街东西段、东湖游园，以及三八路、议台路、文惠街人行道改造等82个城建重点项目，大力推进半截河、东关社区等40个城中村改造项目，完成投资110.5亿元。编制完成许昌区域性交通枢纽规划，加快建设民航异地航站楼，开工建设石武客专许昌东站、长途汽车站等工程，打造综合交通枢纽。全面启动城市数字化管理，深入推进管理精细化和夜景灯饰化，扎实开展交通秩序、广告设置等专项整治，严格控制私搭乱建，提升供水、供气、供热、公交等公用事业服务水平。巩固扩大“四城同创”成果，全面达到国家创模考核要求，扎实推进文明城市创建，致力把许昌建设成为生态宜居的现代化城市。

七、着力保障和改善民生

把保障和改善民生作为发展的重要内容和根本目的，以“十项民生工程”为重点，把惠及民生的事情办得更多、更实、更好。

（一）千方百计扩大就业。实施更加积极的就业政策，以创业带动就业，争创国家级创业型城市。实施全民技能提升工程，全年培训20万人以上。积极推进农村富余劳动力转移就业，促进高校毕业生创业就业，抓好下岗失业人员、就业困难人员再就业。全年新增个体工商户1.5万户，城镇新增就业5万人、农村劳动力新增转移就业8万人、失业人员再就业1.5万人、就业困难人员再就业8000人。加强公共就业服务体系建设，开发更多的公益性就业岗位，动态消除城镇“零就业家庭”，为广大劳动者创造更多的就业机会。

（二）加快发展社会事业。优先发展教育事业，全面推进素质教育，促进义务教育均衡发展。落实市区义务教育学校5年发展规划，合理调整城区中小学校布局，加快推进中小学校舍安全工程，加大农村教师培养培训力度，全面落实“两免一补”，保障农民工子女与城市孩子接受同等教育。大力实施职业教育攻坚计划，推进职教资源整合，加快许昌电气学院、职教园区建设，许昌卫校扩建工程全面启动，许昌陶瓷职业学院首批招生。鼓励社会力量兴办教育，满足群众多样化的教育需求。完善市、县、乡、村四级卫生服务体系，改造5个社区卫生服务中心，建成816所标准化村卫生室，力争2年内全市每个村都有1所标准化卫生室。加强重大传染病防控，提高突发公共卫生事件应急处置能力。大力发展公益性文化事业，加快建设24个乡镇综合文化站、258个农家书屋和851个文化信息资源共享工程村级服务点，公益放映数字电影2.5万场，新增有线电视和地面数字电视3万户。扎实推进体育器材进社区，广泛开展全民健身运动。落实计划生育利益导向政策，稳定低生育水平。

（三）切实加强社会保障。加快构建更加完善的社会保障安全网，使人民生活有基本保障、无后顾之忧。继续扩大养老、医疗、失业、工伤和生育保险覆盖面。城镇居民基本医保和新农合财政补助标准提高到每人每年120元，基本解决关闭破产企业退休人员和困难企业职工的医疗保障问题，基本实现城乡医疗保险全覆盖。抓好新型农村养老保险试点工作。全面提升农村敬老院建设管理水平，巩固集中供养率，实现管理规范化，农村五保年分散供养、集中供养标准分别不低于1200元和2000元。加快实施保障性安居工程，经济适用房新开工34万平方米、竣工30万平方米，建设廉租住房1858套，发放廉租住房租赁补贴10391户，力争2年内基本完成城市棚户区和工矿棚户区改造。完善城乡社会救助体系，大力发展残疾人事业、社会福利和慈善事业。

（四）全力维护社会稳定。开展“安全许昌”创建活动，切实加强煤炭等行业安全生产监管，完成煤炭资源整合重组任务，有效防范和坚决遏制重特大安全事故发生。加强食品药品安全监管，争创食品安全市。加强和改进信访工作，积极化解社会矛盾纠纷。完善应急机制，积极预防、妥善处置群体性事件和突发公共事件。健全社会治安综合防控体系，严厉打击各类违法犯罪，不断提高人民群众的安全感和满意度。加强国防和民兵预备役工作，重视和支持工会、共青团、妇联等群众团体的工作。认真做好第六次人口普查工作。切实做好外事、侨务、对台工作。继续做好民族宗教、地方史志、新闻出版、人事、审计、统计、物价、档案、地震、气象、人防等各项工作。

八、深入推进改革开放

抓重点、求突破，深入推进改革开放，创新科学发展的体制机制，不断增强发展活力和动力。

（一）深化重点领域改革。全面完成市县政府机构改革。注重引进战略合作者，完成39家企事业单位改革改制任务。深化文化体制改革，完成市直专业剧团转企改制。引进社会资本，推进灞陵桥、春秋楼景区改革改制，组建文化旅游集团公司。推进医药卫生体制改革，大力支持社会资本兴办医疗卫生机构，在服务准入、医保定点等方面一视同仁。加快财政投资和融资体制改革，全面推行财政投资评审制度，积极推广项目代建制和回购制，提高市、县两级投融资平台的融资能力。

（二）加大招商引资力度。加快承接境内外产业转移，大力实施“5266”招商引资行动计划，围绕园区、产业、企业、项目和重点区域5个方面，推进20项重大招商活动，重点抓好60个亿元以上招商项目，实现招商引资600亿元。办好第十届中原花木交易博览会、第四届三国文化周和禹州中药材交易会等重大招商活动。在把握规律、转变方式、重在运作中，提高合同履约率、资金到位率、项目开工率，使招商引资工作更富实效。

（三）提升开放型经济水平。优化出口产品和市场结构，巩固发制品出口优势，扩大机电、纺织、高新技术和农副产品出口份额，提高质量和档次，增强国际竞争力。加快企业上市步伐，力争2—3家企业实现上市，争取利用资本市场融资50亿元。深入推进企业战略合作与重组，深化与央企和500强企业的战略合作。积极推进郑许对接，开工建设长葛至新郑城际快速通道，开通许昌至漯河、许昌至平顶山城际公交，加快实现交通一体、联动发展。

九、全面加强政府自身建设

着力提高政府领导科学发展、促进社会和谐的能力和水平，努力建设人民满意的政府。

坚持依法行政。依法接受人大及其常委会的法律监督、工作监督，主动接受政协民主监督，认真办理人大代表建议议案和政协委员提案。严格落实行政执法责任制，加强和改进行政复议，切实做到规范、公正、文明执法。坚持依法、科学、民主决策，使各项决策更加符合许昌实际、更能经得起历史的检验。

坚持为民执政。把更多的公共资源向民生倾斜，向农村、基层和困难群众倾斜。落实好国家调整国民收入分配的各项政策，不断提高城乡居民收入水平。认真解决企业改制、征地拆迁、环境保护、劳动争议、涉法涉诉等领域损害群众利益的突出问题，维护社会公平正义。深入推进政务公开，抓好市长信箱、市长热线、110应急联动服务中心等平台建设，创造条件让群众批评政府、监督政府，让政府的权力在阳光下运行。

坚持高效施政。认真落实“执行力提升年”的要求，深化“五个坚持，两转两提”，坚决杜绝不作为、乱作为、慢作为。坚持按章理事，注重程序规范，继续完善目标管理、新闻发布等工作机制。深化行政审批制度改革，扩大联合审批和网上受理。全面完成电子监察系统建设。加强政风行风评议，严格行政问责，确保政令畅通。大力精减会议和文件，提倡视频会议和网上办公，着力形成清新、简约、责实、务本的良好政风。

坚持廉洁从政。扎实推进惩治和预防腐败体系建设，进一步完善公共资源配置、公共资产交易、公共产品生产等领域的管理制度。加强政府采购管理，推行公务卡制度，深入开展“小金库”专项治理。坚持勤俭行政，反对铺张浪费，大力推进公共机构节能，努力建设节约型机关。充分发挥监察、审计部门的监督作用，严肃查处违法违纪案件，坚决惩处腐败分子，始终做到警钟长鸣、秉公用权、清正廉洁。

各位代表，我们面临的任务艰巨而繁重，我们肩负的责任重大而光荣。让我们在中共许昌市委的正确领导下，深入贯彻落实科学发展观，紧紧依靠、团结带领全市人民，再接再厉，开拓进取，共同创造许昌更加美好的明天！
